# Light-induced shifts in opsin gene expression in the four-eyed fish *Anableps anableps*

**DOI:** 10.3389/fnins.2022.995469

**Published:** 2022-09-29

**Authors:** Daniele Salgado, Bertha R. Mariluz, Maysa Araujo, Jamily Lorena, Louise N. Perez, Rafaela de L. Ribeiro, Josane de F. Sousa, Patricia N. Schneider

**Affiliations:** ^1^Instituto de Ciências Biológicas, Universidade Federal do Pará, Belém, Brazil; ^2^Department of Integrative Biology, Michigan State University, East Lansing, MI, United States; ^3^Department of Biological Sciences, Louisiana State University, Baton Rouge, LA, United States; ^4^Instituto Tecnologico Vale, Belém, Brazil

**Keywords:** evolution, opsin, plasticity, retina, *Anableps*

## Abstract

The development of the vertebrate eye is a complex process orchestrated by several conserved transcriptional and signaling regulators. Aside from partial or complete loss, examples of exceptional modifications to this intricate organ are scarce. The unique eye of the four-eyed fish *Anableps anableps* is composed of duplicated corneas and pupils, as well as specialized retina regions associated with simultaneous aerial and aquatic vision. In a previous transcriptomic study of the *A. anableps* developing eye we identified expression of twenty non-visual and eleven visual opsin genes. Here, we surveyed the expression territories of three non-visual melanopsins genes (*opn4*×*1, opn4*×*2, opn4m3*), one teleost multiple tissue opsin (*tmt1b*) and two visual opsins (*lws and rh2-1*) in dorsal and ventral retinas. Our data showed that asymmetry of non-visual opsin expression is only established after birth. During embryonic development, while inside pregnant females, the expression of *opn4*×*1, opn4*×*2*, and *tmt1b* spans the whole retina. In juvenile fish (post birth), the expression of *opn4*×*1, opn4*×*2, opn4m3*, and *tmt1b* genes becomes restricted to the ventral retina, which receives aerial light. Raising juvenile fish in clear water instead of the murky waters found in its natural habitat is sufficient to change gene expression territories of *opn4*×*1, opn4*×*2, opn4m3*, *tmt1b*, and *rh2-1*, demonstrating that different lighting conditions can shift opsin expression and potentially contribute to changes in spectral sensitivity in the four eyed fish.

## Introduction

Change in environmental light is an important driving force for the diversity of visual systems in vertebrates. Aquatic animals occupy a great range of visual environments and have evolved adaptations to increase sensitivity to the availability of light (Collin, 2009). Evolutionary and developmental adaptations to the visual system have ensured success in avoiding predation, establishing feeding behavior and finding sexual partners (Jeffery, 2020; Thomas et al., 2020). For example, cichlid fishes show a versatile visual system with a complex sensory plasticity that regulates reproductive, feeding, and social behavior (Butler et al., 2019; Maruska and Butler, 2021). Substantial changes to the general structure of the vertebrate eye are rare, and include total or partial loss of the eyes, as seen in cavefishes, and the specialized retinas of the deep-water fish (Warrant and Locket, 2004; Biagioni et al., 2016; Lunghi et al., 2019).

**FIGURE 1:**
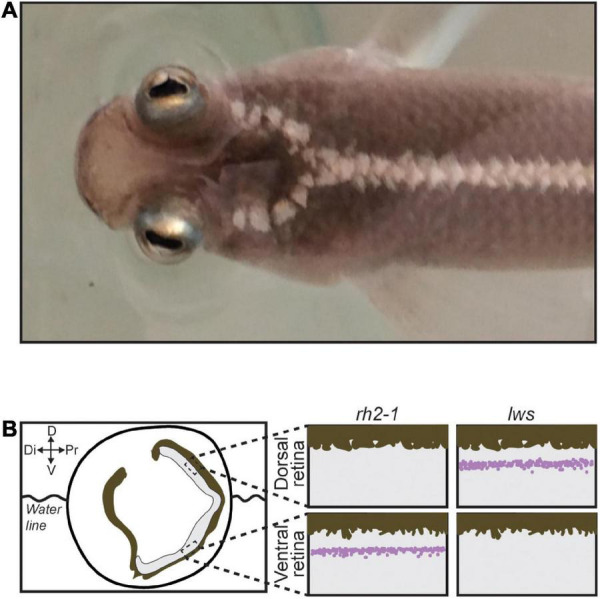
The *Anableps* anableps. **(A)** Anableps anableps female specimen, dorsal close-up view of the *A. anableps* head and part of the body. **(B)** Schematic of the *A. anableps* eye showing the water line and asymmetric gene expression of *lws* and *rh2-1* genes in the dorsal and ventral retinas, respectively. Dorsal (D), Di (Distal), V (Ventral), and Pr (Proximal).

Attempt in eye duplication is a rare event observed in only a few fish lineages such as *Dialommus fuscus* and *Dialommus macrocephalus*, also known as “Rockskipper,” these fish are terrestrial predators and have a vertically split cornea that allows them to remain upright with one cornea in and the other out of the water. In addition, *Bathylychnops exilis*, inhabits depths between 600 and 3,000 ft, and has an auxiliary globe in addition to the main eyeball directed toward the ocean floor (Schwab et al., 2001). Species from the genus *Anableps* are also a remarkable example of extreme adaptation of the visual system. With a partially duplicated eye, simultaneous aerial and aquatic vision, and a single optic nerve, *Anableps* consists in a unique model to understand the genetic bases of eye duplication (Figure 1A; Schwab et al., 2001; Owens et al., 2012; Perez et al., 2017).

Morphological innovations to the visual system require molecular changes in photoreception regulation. The distribution of photoreceptor cells and the composition of rods and cones play an important role in spectral sensitivity in teleost fish (Okano et al., 1994; Collin, 2009; Sato et al., 2018).

Membrane proteins known as visual opsins are present in retina cells and act as light sensors. Coupled with chromophores, these proteins mediate light detection and the initiation of visual processing in the photoreceptor cells (Wald, 1968; Yokoyama, 2000). Conversely, non-visual opsins, although expressed in the inner retina among other tissues in the vertebrate body, do not participate directly in image formation and instead respond to light by activating a series of non-visual sensory responses. In fish, these proteins are present in the pineal complex, epidermal photoreceptors and in the retina nuclear layers and play an important role in light detection, neural development and control of the circadian rhythm (Okano et al., 1994; Kojima and Fukada, 1999; Van Gelder, 2001; Peirson et al., 2009).

The melanopsins are the most widely studied non-visual opsins (Peirson et al., 2009; Diaz et al., 2016; Van Gelder and Buhr, 2016; Guido et al., 2020). In zebrafish (*Danio rerio*), expression of *opn4*×*1a*, *opn4*×*1b*, and *opn4m3* are detected in horizontal, bipolar and amacrine cells while a small subgroup of retinal ganglion cells expresses *opn4*×*1* and *opn4m3*. The expression of *opn4m3* was also detected in the zebrafish retinal pigment epithelial (RPE) (Davies et al., 2011). In the retina cells of salmon fish (*Salmo salar*), five melanopsin genes were observed: *opn4*×*1a* and *opn4*×*1b*, *opn4m1a1*, *opn4m1a2*, and *opn4m2* (Sandbakken et al., 2012). The non-visual opsin multiple tissue of teleosts (*tmt*) is expressed in the eyes, brain, liver, kidneys, and heart of fugu (*Fugu rubripes*) and zebrafish and in the brain and eyes of medaka (*Oryzias latipes*) (Moutsaki et al., 2003; Fischer et al., 2013). Previous studies have demonstrated that tmt1 and tmt2 opsins act as a blue light-sensitive Gi/Go-coupled receptors but exhibited spectral properties and photo-convertibility of the active state different from each other (Diaz et al., 2016; Guido et al., 2020). While non-visual opsins are expressed in the retina cells of many fish species, the exact role of these proteins and their physiological functions remain to be elucidated (Diaz et al., 2016; Guido et al., 2020).

Asymmetric gene expression of opsin genes is another way of modulating visual sensitivity, and this mechanism plays an important role in the diversification of visual pigments in fish, leading to changes in spectral sensitivity (Hofmann and Carleton, 2009; Hauser and Chang, 2017). In addition, modulation of spectral sensitivity could also occur as part of a short-term plastic response to environmental changes, as observed in the eyes of the four eyed fish *Anableps anableps.*

*Anableps* are livebearers; female fish give birth to fully developed progeny, similarly to mammals. Previous work from our group has shown that the establishment of asymmetric expression of visual opsins (*rh2-1* and *lws*) occurs prior to birth and is independent of photic input, suggesting that it is genetically programmed (Perez et al., 2017). Here, we show that *opn4*×*1*, *opn4*×*2*, and *tmt1b* are expressed symmetrically in the entire retina of the developing eye of the *A. anableps* during larval stages. In the juvenile fish, *tmt1b, opn4*×*1, opn4*×*2*, and *opn4m3* shift the expression domain and become restricted to the ventral retina. *A. anableps* inhabit murky waters of the Amazon River and its tributaries (Supplementary Video 1). However, when animals were maintained in aquaria with clear water, we observed loss of *opn4*×*2* expression in the retina, and a shift in *opn4*×*1*, *opn4m3*, and *tmt1b* expression domains, from being restricted to the ventral retina, to being expanded dorsally and occupying the entire retina, recapitulating the expression pattern of the developing eye during larval stages.

## Materials and methods

### Sample collection

Specimens of *A. anableps* were collected by seine net in two locations: Abaetetuba, Pará, Brazil (1°42′24.9″S 48°53′46.4″W) and Bragança, Pará, Brazil (0°53′27.6″S 46°39′21.9″W). Animals were euthanized using MS-222 (Sigma-Aldrich Corp., Milwaukee, WI, USA) followed by decapitation.

### Histological analysis

Eyes of wild-caught *A. anableps* juvenile (up to 15 cm long) and larvae were collected and flash frozen in Tissue-Tek^®^ O.C.T TM embedding medium (Sakura Finetek USA Inc., Torrance, CA, USA). Sagittal cryosections (20 μm) of the eyes were obtained using CM1850 UV cryostat (Leica Biosystems Nussloch GmbH, Nussloch, BW, Germany) and collected on ColorFrost Plus microscope slides (Thermo Fisher Scientific, Pittsburgh, PA, USA), fixed in 3% paraformaldehyde (Sigma-Aldrich Inc., St. Louis, MO, USA) and stored at −80°C for further use.

### Lighting environment conditions

Eight juvenile fish were kept in a 75 Gallon glass aquarium with UV filter and standard fluorescent light (40 watts); fish were kept in dechlorinated tap water at 28°C with aeration and biological and mechanical filtration and fed daily with fish ration. Fish were exposed to a day and night cycle of 12 h for 30 days followed by euthanasia, according to Fuller and Claricoates (Fuller and Claricoates, 2011; Supplementary Video 2).

Sagittal sections (20 μm) were obtained using CM1850 UV cryostat (Leica Biosystems Nussloch GmbH, Nussloch, BW, Germany), placed on ColorFrost Plus microscope slides (Thermo Fisher Scientific, Pittsburgh, PA, USA) and stored at −80°C for further use. Eight wild-caught juvenile fish were used as control for these experiments.

### Riboprobe synthesis

To produce riboprobes for *in situ* hybridization for the genes *opn4m3*, *opn4*×*1*, *opn4*×*2*, and *tmt1b*, we adapted a PCR-based technique for the preparation of riboprobe templates (David and Wedlich, 2001). First, vectors containing target gene sequences were synthesized in pBlueScript II SK(+) by BioCat GmbH (Heidelberg, Germany). Next, these vectors were used as a template on a PCR reaction. The T7 promoter sequence was included at the 5′ end of each gene-specific forward primer, whereas the SP6 promoter sequence was added to the 5′ end of each gene-specific reverse primer. The resulting amplicons for each target gene were subsequently used as template for sense (T7) or antisense (SP6) riboprobe synthesis. Primers (with promoter sequences underlined) were: *opn4m3*-Forward: 5′ TAATACGACTCACTATAGGCTCGTCTGAG GTGGTTTTAGG 3 and reverse: 5′ ATTTAGGTGACAC TATAGTGTAGGAGCAGGGTGGAGAAG 3′, *opn4*×*1*-Forward: 5′TAATACGACTCACTATAGCAAAGAAGGCAAC GATGTAGTGA 3′ and reverse: 5′ ATTTAGGTGACACTA TAGACACGGATTCTATCGGCATGT 3′, *opn4*×*2*-Forward: 5′ TAATACGACTCACTATAGCCATTCCTTGTAGAGGCAGTT GATA 3′ and reverse: 5′ ATTTAGGTGACACTATAGGCGACT TCCTCATGGCTTTC3′, *tmt1b*-Forward: 5′ TAATACGA CTCACTATAGCAGTGGTCGTCCGAAAATGG 3′ and reverse: 5′ ATTTAGGTGACACTATAGGCGGTTCAAAGGG ATTACTGT 3′.

To produce riboprobes for the visual opsins *lws* and *rh2-1*, we used as templates vectors previously described (Perez et al., 2017). The pCRII-TOPO vector (Thermo Fisher Scientific, Pittsburgh, PA, USA) containing the target gene sequence was used as a template in a PCR reaction with M13 forward (5′ TAAAACGACGGCCAG-3′) and M13 reverse (5′-CAGGAAACAGCTATGAC 3′) primers. The resulting amplicon contained the sequence of the gene of interest flanked by an upstream T7 promoter and a downstream SP6 promoter. Sense and antisense probes were produced using T7 and SP6 RNA polymerases, respectively. Riboprobe reaction was performed using DIG-labeling mix (Roche Diagnostics Deutschland GmbH, Mannheim, BW., GER.) and according to manufacturer’s protocol (mMESSAGE mMACHINE Transcription kits, Ambion, Carlsbad, CA, USA).

### *In situ* hybridization

*In situ* hybridization was performed according to previously established protocol (Lovell et al., 2013; Carleton et al., 2014; De Lima et al., 2015). Cryosections from heads *A. anableps* specimens were acetylated for 10 min in a solution of 0.33% acetic anhydride in DEPC-treated water, rinsed with 2× SSPE buffer (0.02 M EDTA and 2.98 M NaCl in 0.2 M phosphate buffer) and dehydrated in 70, 95, and 100% RNase-free ethanol. Each section was then hybridized with a solution (32 μL) containing 50% formamide, 2× SSPE, 1 μg/μl BSA, 1 μg/μL poly(A) (Sigma-Aldrich Corp., Milwaukee, WI, USA) in DEPC-treated water, and 1 μL of the DIG-labeled riboprobe. Slides were cover slipped, sealed by immersion in mineral oil, and incubated overnight at 65°C. Next, sections were rinsed in chloroform, de-cover slipped in 2× SSPE, and incubated serially in 2× SSPE/50% formamide, and in 0.1× SSPE at 65°C. Sections were then blocked for 30 min at RT in blocking buffer with 1% skim milk and incubated overnight with an alkaline phosphatase-conjugated anti-DIG antibody (Roche Diagnostics Deutschland GmbH, Mannheim, BW, Germany) Slides were washed twice for 15 min in TMN (100 mM Tris, pH 9.5; 150 mM NaCl, 0.05 M MgCl2), and incubated in a detection solution containing the alkaline phosphatase substrates Nitro-Blue Tetrazolium Chloride (NBT) (Roche Diagnostics Deutschland GmbH, Mannheim, BW, Germany). and 5-Bromo-4-Chloro-3-Indolyl-phosphate p-Toluidine Salt (BCIP) (Roche Diagnostics Deutschland GmbH, Mannheim, BW, Germany) Slides were washed overnight, fixed in 4% paraformaldehyde (dissolved in PBS) and cover slipped with Cytoseal (Thermo Fisher Scientific, Pittsburgh, PA, USA). Slides were imaged using a Nikon SMZ1500 microscope and processed on NIS-Elements D 4.10.01 program (Nikon Instruments Inc., Melville, NY, USA).

## Results

### Non-visual opsins are expressed symmetrically in the developing eye of *Anableps anableps* prior to birth

**FIGURE 2:**
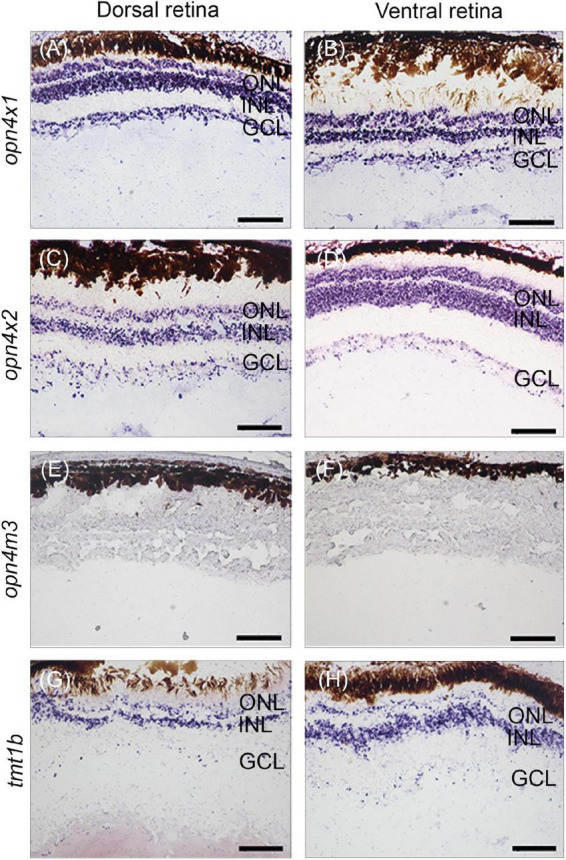
Non-visual opsin gene expression in the larval retina of *Anableps anableps*. *In situ* hybridizations for o*pn4×1* **(A,B)**, *opn4×2* **(C,D)**, *opn4m3* **(E,F)**, and *tmt1b* **(G, H)** were performed in eyes of larvae collected before birth. Sections of the dorsal retina are on the left, and ventral retina to the right. ONL, outer nuclear layer; INL, inner nuclear layer; GCL, ganglion cell layer. Scale bars: 0.05 mm.

Our previous work identified twenty non-visual and eleven visual opsins expressed during the development of the eye in the four-eyed fish (Beaudry et al., 2017; Perez et al., 2017). Here, *in situ* hybridization was performed to examine the expression domains of three types of melanopsin subfamily of genes, *opn4*×*1*, *opn4*×*2*, *opn4m3*, and one type of teleost multiple tissue opsin, *tmt1b*, in the developing retina of pre-birth larvae. Our results showed that *opn4*×*1, opn4*×*2, and tmt1b* are expressed uniformly in the outer nuclear layer (ONL), inner nuclear layer (INL), and ganglion cell layer (GCL) in both dorsal and ventral regions of the retina (Figures 2A–D,G,H). The expression of *opn4m3* was not detected by *in situ* hybridization at stage 5 (Figures 2E,F).

### Expression domains of visual and non-visual opsins change upon different lighting conditions in juvenile *Anableps anableps*

We have previously shown that in the *A. anableps*, asymmetric expression of *lws* and *rh2.1* in the dorsal and ventral regions of the retina, respectively, is established prior to birth in photoreceptor cells (Perez et al., 2017). Here, we wanted to investigate the plasticity of visual and non-visual opsins in the retina upon changes in environmental lighting. To this end, juvenile fish were maintained in clear water for 30 days and surveyed for visual and non-visual opsin gene expression using RNA *in situ* hybridization.

**FIGURE 3:**
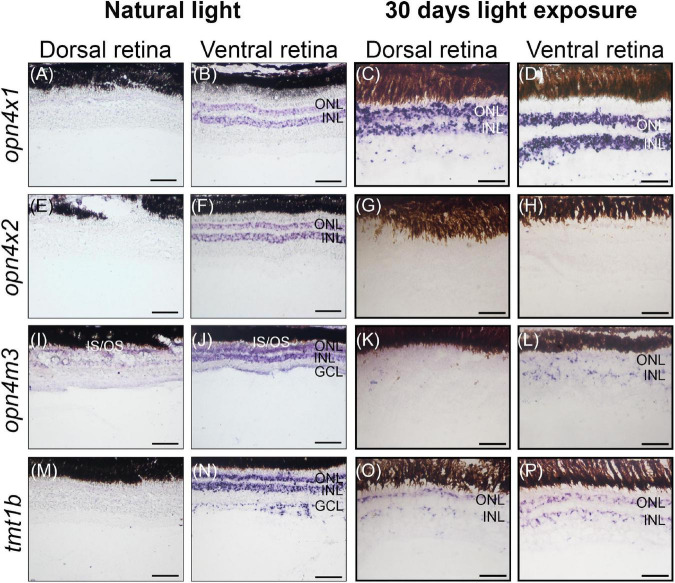
Non-visual opsin gene expression in the juvenile retina of *Anableps anableps*. *In situ* hybridizations of *opn4×1* **(A–D)**, *opn4×2* **(E–H)**, *opn4m3* **(I–L)**, and *tmt1b* **(M–P)** were performed in *A. anableps*’ eyes from wild-caught juvenile fish under natural light conditions **(A,B,E,F,I,J,M,N)** and juveniles that were kept for 30 days in light exposure from above and below the water **(C,D,G,H,K,L,O,P)**. The panel shows sections of the dorsal retina and ventral retina. ONL, outer nuclear layer; INL, inner nuclear layer; GCL, ganglion cell layer; IS, photoreceptor inner segments; OS, photoreceptor outer segments. Scale bars: 0.05 mm.

In wild-caught juvenile *A. anableps* found in murky river water, the expression of *opn4*×*1* (Figures 3A,B) and *opn4*×*2* (Figures 3E,F) is restricted to the ONL and INL of the ventral retina while *opn4m3* and *tmt1b* are expressed in the INL, ONL and GCL of the ventral retina (Figures 3I,J,M,N). We next used a 30-day light-induced cycle and assayed opsin gene expression. Our results showed that expression of *opn4*×*1* and *tmt1b*, restricted to the ONL and INL in the ventral retina in wild-caught fish, expands to the ONL, INL and GCL in the dorsal retina (Figures 3C,D,O,P); *opn4*×*2* expression is lost in the entire retina (Figures 3G,H) while *opn4m3* expression becomes sparse in the ONL, INL and GCL of the ventral retina (Figures 3K,L).

**FIGURE 4:**
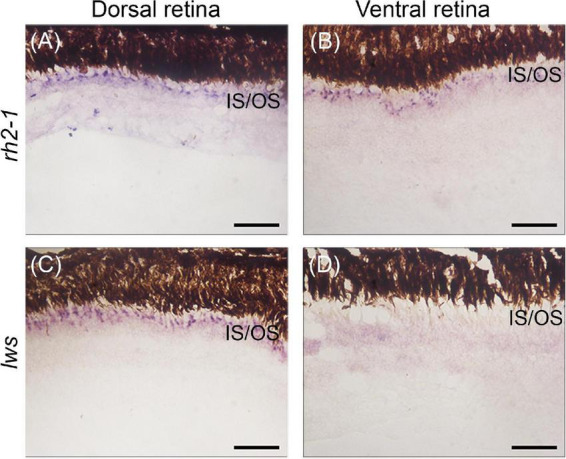
Gene expression of visual opsins in the retina of juvenile *A. anableps* under light stimuli for 30 days. *In situ* hybridizations of *rh2-1* **(A,B)** and *lws* **(C,D)** in dorsal and ventral retinas from juveniles of *A. anableps*. Scale bars: 0.05 mm.

We next assayed the *lws* and *rh2-1* visual opsins, which show expression domains restricted to the dorsal and ventral retinas, respectively (Perez et al., 2017). Upon light-induced exposure, *in situ* hybridization showed that *lws* expression remained in the photoreceptor layer of the dorsal retina, however, *rh2-1* expression expanded to the photoreceptor layer of ventral and dorsal retina territories (Figures 1B, 4A–D for reference).

Altogether, our results show that *lws* and *rh2-1* visual opsin expression is established without environmental light stimuli, prior to birth. However, changes in light input can modulate the expression of *rh2-1* and cause a shift the expression domain, from being restricted to the ventral retina, to being expanded to the entire retina. Non-visual opsins also show asymmetric expression in the nuclear layer of the retina in juvenile fish. Although established only after birth, changes in the environmental lighting caused a shift in the expression domains of *opn4*×*1*, *opn4m3*, and *tmt1b*, suggesting that both visual and non-visual opsins can be modulated by changes in the photic input.

## Discussion

In this work, we surveyed the expression of three melanopsins *opn4*×*1*, *opn4*×*2, opn4m3*, and one *tmt1b* in the nuclear layers of the *A. anableps* retina. The spatial pattern observed pre-birth diverges from that seen in free-swimming juvenile individuals, which express *opn4*×*1* and *opn4*×*2* in the ONL and INL of the ventral retina while *opn4m3* and *tmt1b* are expressed in the INL, ONL and GCL of the ventral retina.

In zebrafish, melanopsin genes are expressed in different regions of the retina and have been associated with the regulation of negative phototaxis (Matos-Cruz et al., 2011; Fernandes et al., 2012). In teleost fish, the expression of melanopsin in various types of neuronal cells can directly influence retinal function in a dependent and/or independent way from intrinsically photosensitive retinal ganglion cells (Matos-Cruz et al., 2011). In other vertebrates, including teleost fish, *tmt* expression is distributed across many tissues including internal organs where light can hardly penetrate, suggesting that *tmt* opsin may have both photosensitive and non-photosensitive functions (Fischer et al., 2013; Sakai et al., 2015). In our work, we speculate that light-induced changes in non-visual opsins expression could be due to the indirect regulation of visual opsins in the photoreceptor cells of the retina.

Previous studies from our group and others have shown asymmetric expression of *lws* and *rh2.1* in the photoreceptor cells in the dorsal and ventral domains of the developing retina, respectively, with the establishment of asymmetric expression of *lws* and *rh2.1* occurring before birth, suggesting that it is genetically programmed and independent of light stimuli (Owens et al., 2012; Perez et al., 2017). This asymmetry allows the partial duplicated eye of the *A. anableps* to optimize the absorption of different wavelengths coming simultaneously from the aerial and aquatic environment. In this work, we manipulated light input by changing the turbid water where the *A. anableps* is normally found and replacing it with clear water. We tested the plasticity of *lws* and *rh2.1* in the photoreceptor cells in the retina of juvenile fish after a 30 day period and our results showed that, despite being established before light stimuli reach the retina (Perez et al., 2017), the expression of *rh2.1* (normally expressed in the ventral retina) shifted and became uniformly expressed in the entire retina, while the expression of *lws* remained unchanged and restricted to the photoreceptor cells in the dorsal retina. While in captivity, animals were maintained in tanks with average temperature of 28°C, which closely mirrors that of their natural environment. The commercial diet provided for the animals in captivity, however, differs from their diet in natural settings. In the wild, the stomach content of *A. anableps* ranges from micro and macroalgae, insects, crustaceans, molluscs, fishes, to mud and detritus (Figueiredo et al., 2019). While we cannot rule out a potential feeding behavior of food content impact on opsin gene expression, we have no knowledge of a documented link between the former and the latter.

In zebrafish and guppies, the expression patterns of different subtypes of *rh2* and *lws* vary according to the developmental stage and region of the retina (Takechi and Kawamura, 2005; Owens et al., 2012). Several studies have demonstrated that opsin expression is subject to phenotypic plasticity (Fuller et al., 2005; Shand et al., 2008; Veen et al., 2017). Several teleost species placed under altered light conditions, such as damsel and cardinal fish (Luehrmann et al., 2018), African cichlids (Wright et al., 2019), guppies (Kranz et al., 2018), and Senegalese sole (Frau et al., 2020) can alter the expression of opsin genes to adapt to the environmental changes. Here we show that shift in opsin expression happens in the dorsal and ventral domains of the retina in *A. anableps* within a 30-day period, our results do not exclude the possibility that the change in the expression pattern of opsin genes occurs in a shorter period. Here, we show that after a 30-day period of exposure to clear water, opsin expression changes in the dorsal and ventral retinas, given that, this shift happens in portions of the retina for both visual and non-visual opsin genes, our results suggest that the regulatory control of the expression of opsins in the eye of the *A. anableps* is complex and compartmentalized in the dorsal and ventral retinas.

## Conclusion

Our study shows that the expression of visual opsins in the retina of *A. anableps* can be modulated by spectral and intensity changes in ambient light. These adjustments allow for a quick adaptation of the visual system to different water turbidity and environmental luminosity. Our findings indicate that the *A. anableps* exhibits great potential to modulate its vision according to the environment, allowing this organism to adjust its vision to different photic conditions. Furthermore, our findings suggest that the expression of non-visual opsins can also be altered upon changes in environmental light, suggesting a regulatory correlation between visual and non-visual opsin regulation of physiological functions responsive to light.

## Data availability statement

The raw data supporting the conclusions of this article will be made available by the authors, without undue reservation.

## Ethics statement

This study was approved by IBAMA/SISBIO under license number 47206-1 and the Ethics Committee for Animal Research at the Universidade Federal do Pará (protocol number 037-2015).

## Author contributions

DS, MA, JL, LP, and PS designed the research. LP, MA, JL, and BM performed *in situ* hybridizations (ISH) of *A. anableps* larvae. RR and JS designed the riboprobes for ISH. DS and LP performed light-induced experiments and analysis. DS, LP, and PS wrote the manuscript with input from BM, MA, JL, RR, and JS. All authors contributed to the article and approved the submitted version.

## Supplementary material

Supplementary Figure 1 *In situ* hybridization showing the sense control riboprobes for the non-visualopsins: *opn*4×1, *opn*4×2, *opn4m3*, *tmt1b*.

Supplementary Figure 2 *In situ* hybridization showing the sense control riboprobes for thevisual opsins *lws* and *rh2-1*.

Supplementary Video 1 A group of five *Anableps anableps* specimens in the wild. Movie was recorded in the estuarine region of Bragança, Pará, Brazil (0°53′27.6″S 46°39′21.9″W).

Supplementary Video 2 A fish tank with *Anableps anableps* specimens during the 30-day light induced experiments.
