# Supplementary figures and images for: Light-induced shifts in opsin gene expression in the four-eyed fish Anableps anableps
Source: Front Neurosci. 2022 Sep 29;16:995469. doi: 10.3389/fnins.2022.995469 (PMC9556854; doi:10.3389/fnins.2022.995469)

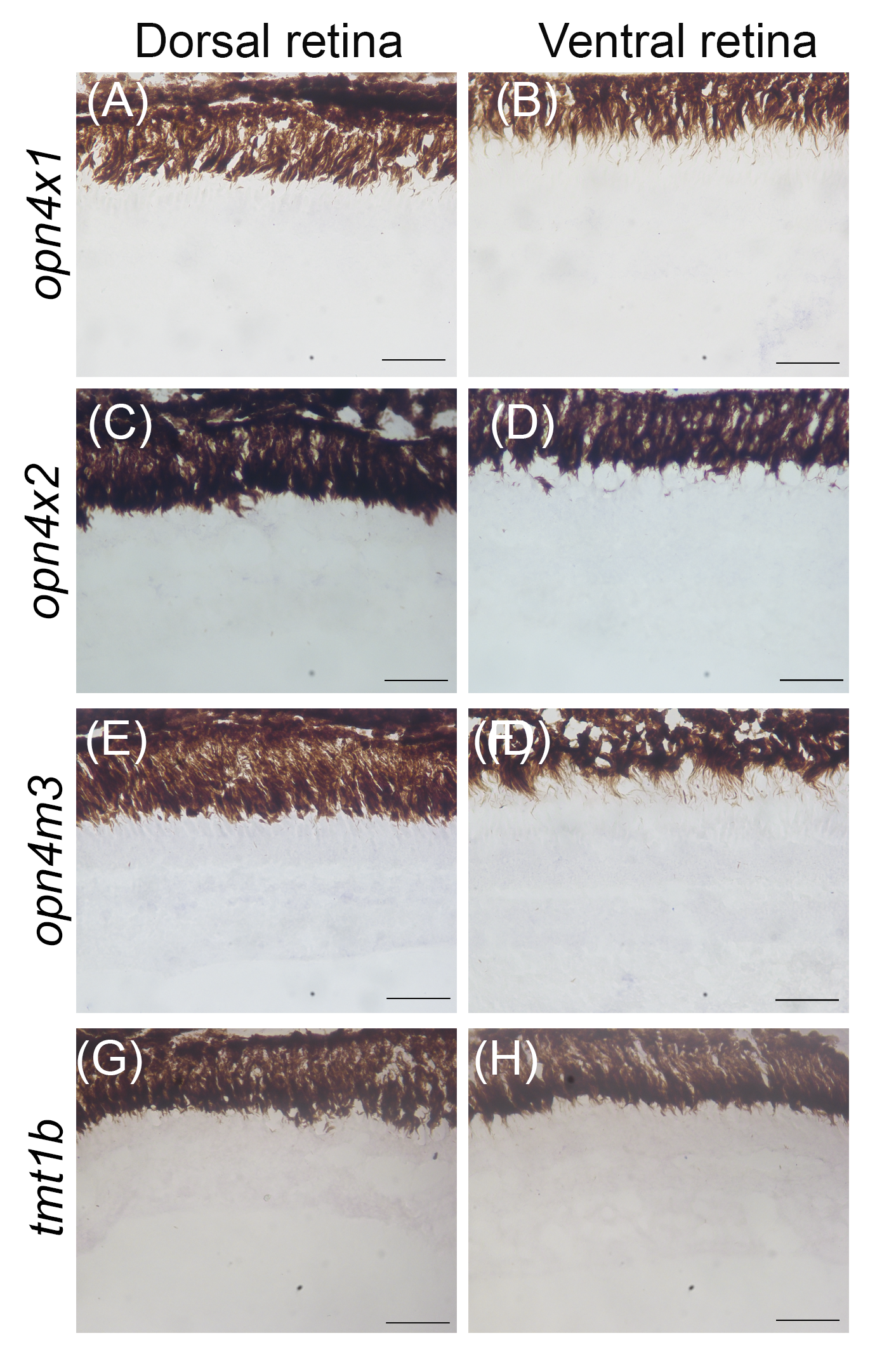

Supplement: Supplementary Figure 1 — In situ hybridization showing the sense control riboprobes for the non-visualopsins: opn4×1, opn4×2, opn4m3, tmt1b. [file Image_1.TIF]

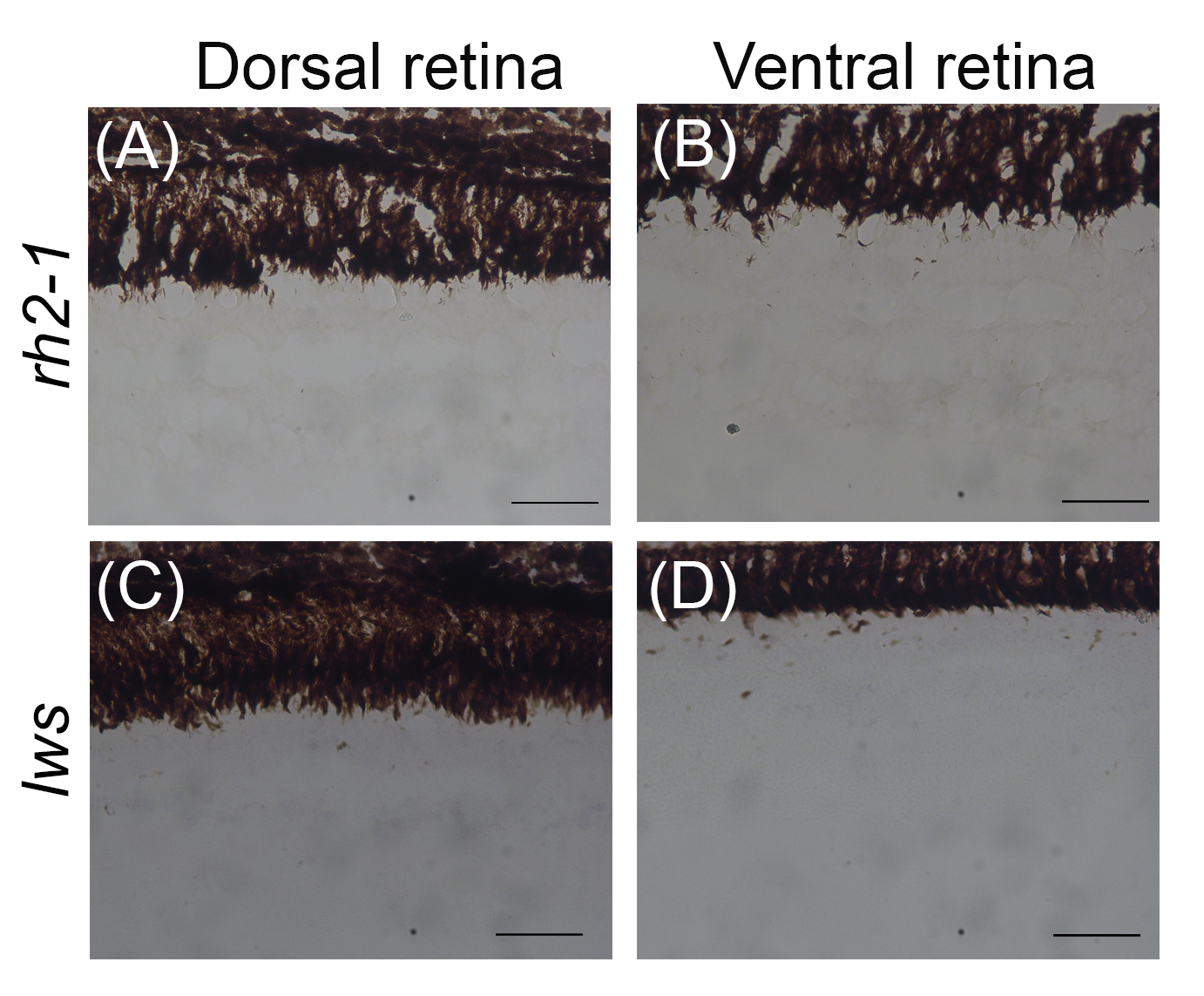

Supplement: Supplementary Figure 2 — In situ hybridization showing the sense control riboprobes for thevisual opsins lws and rh2-1. [file Image_2.TIF]
